# Transmural and rate-dependent profiling of drug-induced arrhythmogenic risks through in silico simulations of multichannel pharmacology

**DOI:** 10.1038/s41598-019-55032-x

**Published:** 2019-12-06

**Authors:** Ping’an Zhao, Pan Li

**Affiliations:** 1 Center for Public Health Informatics, School of Public Health, Xinxiang Medical University, Henan, P.R. China; 2 Center for Biomedical Innovation, Yunmai Biomedical Research Institute, Henan, P.R. China

**Keywords:** Cardiology, Diseases, Medical research

## Abstract

*In vitro* human ether-à-go-go related gene (hERG) inhibition assay alone might provide insufficient information to discriminate “safe” from “dangerous” drugs. Here, effects of multichannel inhibition on cardiac electrophysiology were investigated using a family of cardiac cell models (Purkinje (P), endocardial (Endo), mid-myocardial (M) and epicardial (Epi)). We found that: (1) QT prolongation alone might not necessarily lead to early afterdepolarization (EAD) events, and it might be insufficient to predict arrhythmogenic liability; (2) the occurrence and onset of EAD events could be a candidate biomarker of drug-induced arrhythmogenicity; (3) M cells are more vulnerable to drug-induced arrhythmias, and can develop early afterdepolarization (EAD) at slower pacing rates; (4) the application of quinidine can cause EADs in all cell types, while I_NaL_ is the major depolarizing current during the generation of drug-induced EAD in P cells, I_CaL_ is mostly responsible in other cell types; (5) drug-induced action potential (AP) alternans with beat-to-beat variations occur at high pacing rates in P cells. These results suggested that quantitative profiling of transmural and rate-dependent properties can be essential to evaluate drug-induced arrhythmogenic risks, and may provide mechanistic insights into drug-induced arrhythmias.

## Introduction

Drug-induced cardiotoxicity has been a major concern since the early stage of novel drug development. Unexpected post-marketing occurrence of cardiotoxic effects remains a leading cause of drug withdrawal and relabelling^1–3^. As defined by the International Conference of Harmonization Expert Working Group for all drugs in development, QT interval prolongation has been used as a biomarker to predict the potential risk of Torsade de Pointes (TdP)^4,5^. Most drugs that prolong the QT interval inhibit cardiac potassium channels encoded by human ether-à-go-go related gene (hERG); therefore, the level of hERG channel inhibition has been the “gold standard” to predict the TdP risk. However, recent studies suggested that the *in vitro* hERG blockade assay alone provides insufficient information to accurately discriminate “safe” from “dangerous” drugs^6^. For instance, QT prolongation can be induced by drugs that inhibit other ion channels, such as the slow inward-rectifier K^+^ channel (encoded by KCNQ1 and KCNE1^7^); the arrhythmia associated with hERG inhibition can be mitigated by concurrent blockade of inward Na^+^ or Ca^2+^ currents^8,9^. As suggested by Kramer *et al*.^10^, *in vitro* assays that evaluate multiple ion channel effects (Nav1.5, Cav1.2 and hERG) may improve the prediction of TdP risks when compared to the hERG assay. More recently, the Comprehensive *in vitro* Proarrhythmia Assay (CiPA) has been proposed to address the misidentification issue of drug-associated TdP risk based on hERG inhibition and QT prolongation data. This new paradigm is based on integrated assessment of a wider range of ion channel dynamics (including Nav1.5 (peak and late), Cav1.2, hERG, Kv4.3, KCNQ1/KCNE1, Kir2.1) in delayed ventricular repolarization; alterations to this process lead to repolarization instability and arrhythmias^11^. In addition to drug-induced QT prolongation, action potential (AP) triangulation has also been reported as a risk factor for both atrial fibrillation and ventricular arrhythmia^12^. Furthermore, the autonomic nervous system can also play an important role in drug-induced TdP risks^13^, and as proposed by Champeroux *et al*.^14^, the high frequency relationship between magnitude of heart rate and QT interval changes may serve as a new biomarker for assessing TdP liability.

Multi-scale modelling of the heart has been an important tool in advancing our understanding of cardiac excitation-contraction coupling under both physiological and pathological conditions^15^. Recent pharmacological studies have utilized cardiac cell models for the *in silico* evaluation of drug-induced proarrhythmic risks^11,16–18^. For example, multiple cardiac ion channels were integrated into the human ventricular cell model to improve the assessment of proarrhythmic risk^19,20^. Lancaster *et al*. proposed a computational approach that combines a human ventricular myocyte model of drug effects and machine learning to evaluate drug-induced TdP risks^21^. Using *in silico* human endocardial and Purkinje cell models, Le Guennec *et al*. evaluated how intra- and inter-individual variability can influence drug-induced proarrhythmic effects of hERG inhibition^16^. In addition, tissue models of the heart have also been implemented to quantitatively evaluate drug-induced risk^22^, e.g. Kubo *et al*. developed a 2-dimensional ventricular wedge preparation model of both non-failing and failing hearts to predict drug-induced QT prolongation and ventricular proarrhythmia^23^.

The mammalian heart functions as a complex biological system^24^, and its emergent properties (e.g. TdP) mostly result from the interplay of sub-system components (e.g. Purkinje cells, and various types of cardiac myocytes across the ventricular wall)^25^. Here, we employed a family of *in silico* cardiac cell models (Purkinje(P), endocardial(Endo), mid-myocardial(M) and epicardial(Epi)) and performed quantitative profiling of drug-induced arrhythmogenic risk at physiological pacing frequencies, to identify potential novel characteristics associated with drug-induced TdP risk, and to provide possible mechanistic insights into drug-specific cardiotoxicity that may facilitating the process of drug development.

## Results

### Transmural heterogeneity of AP morphologies and APD rate adaptations

**Figure 1:**
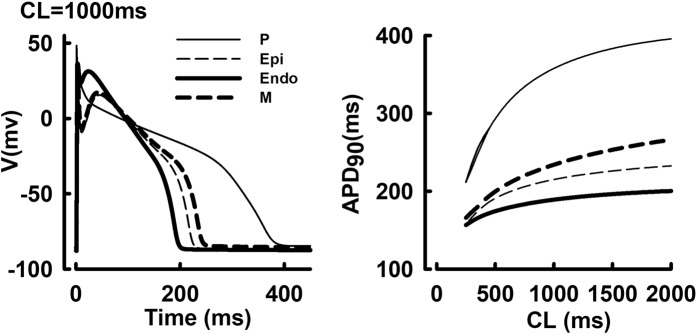
Steady-state Transmural AP morphologies (CL = 1000 ms) (**A**) and APD rate adaptation curves (**B**) in P, Endo, M and Epi cells.

Transmural AP morphologies (steady-state pacing at cycle length (CL) = 1000 ms) and AP duration (APD) rate adaptation curves are shown in Fig. 1. The APD in M cells was longer than that in Epi or Endo cells at all CLs, yet was considerably shorter than the APD in P cells. The AP amplitude (APA) in P cells was higher than that in ventricular cells. In Epi and M cells, AP reproduced a characteristic phase-1 notch and dome morphology, which was not apparent in Endo cells. The APD rate adaptation curve is much steeper at short CLs in P cells, compared to ventricular cells. These simulated AP morphologies and APD adaptation curves were consistent with earlier experimental measurements^26,27^.

### Drug-induced changes in APD rate adaptations

**Figure 2:**
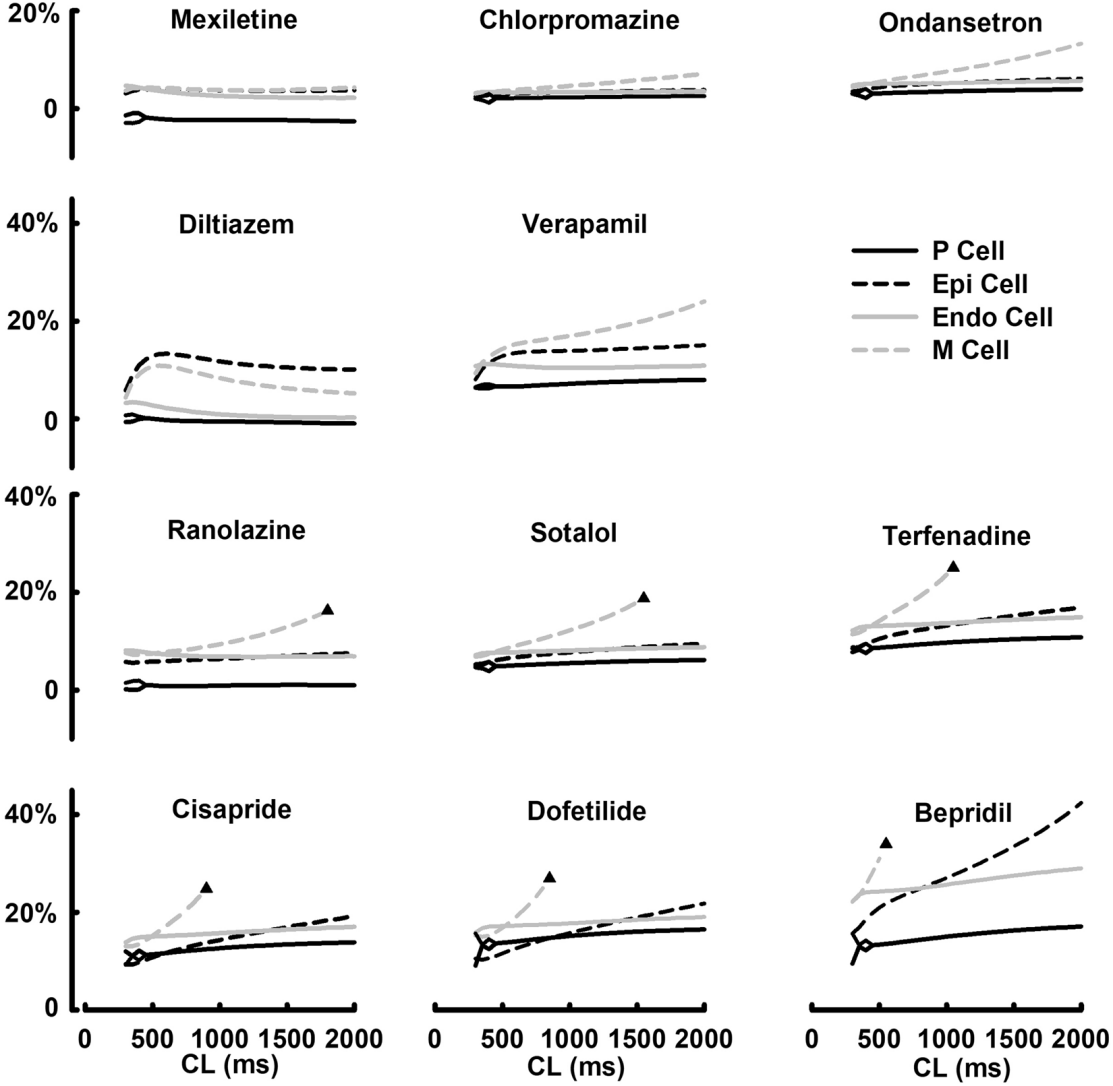
Steady-state drug-induced changes in APD rate adaptations in P, Endo, M and Epi cells. All drugs were applied at their effective free therapeutic plasma concentrations (EFTPCs). Black triangles indicate the onset of EAD events.

Drug-induced changes in APD rate adaptations were shown in Fig. 2, with a detailed summary of drug-induced APD prolongation, EAD and AP alternans provided in the Supplemental Table S1. With the application of qunidine, early afterdepolarization (EAD) events were observed in all cell types at all pacing CLs. With the application of ranolazine, sotalol, terfenadine, cisapride, dofetilide or bepridil, EAD events were observed at different CLs, exclusively in M cells. Bepridil can induce EAD at CL >550 ms, while ranolazine can induce EAD at CL >1800 ms. Major drug-induced APD prolongation (>10%) was observed with the application of bepridil, dofetilide, cisapride or terfenadine, while ranolazine or sotalol caused moderate APD prolongation. With the application of diltiazem or verapamil, major APD prolongation can be observed without EAD events, suggesting APD prolongation alone could be insufficient to trigger EAD. Chlorpromazine or ondansetron caused moderate prolongation without EAD events. In contrast, mexiletine led to minor APD shortening by 2.6% at CL = 2000 ms in P cells, while causing minor APD prolongation in other cell types with no EAD events. In general, the percentages of drug-induced APD prolongations were much higher in M cells. For example, at CL = 1000 ms, bepridil caused APD prolongation of 15% (P cells), 27% (Epi cells) and 25.6% (Endo cells) respectively, and caused APD prolongation of 158% in M cells (with EAD). Drug-induced changes in APD is highly cell- and rate-dependent. For example, drug-induced EAD events are mostly associated with slower pacing rates in M cells; drug-induced AP alternans mostly occur at fast pacing rates in P cells.

### Transmural variations of drug-induced arrhythmogenicity

**Figure 3:**
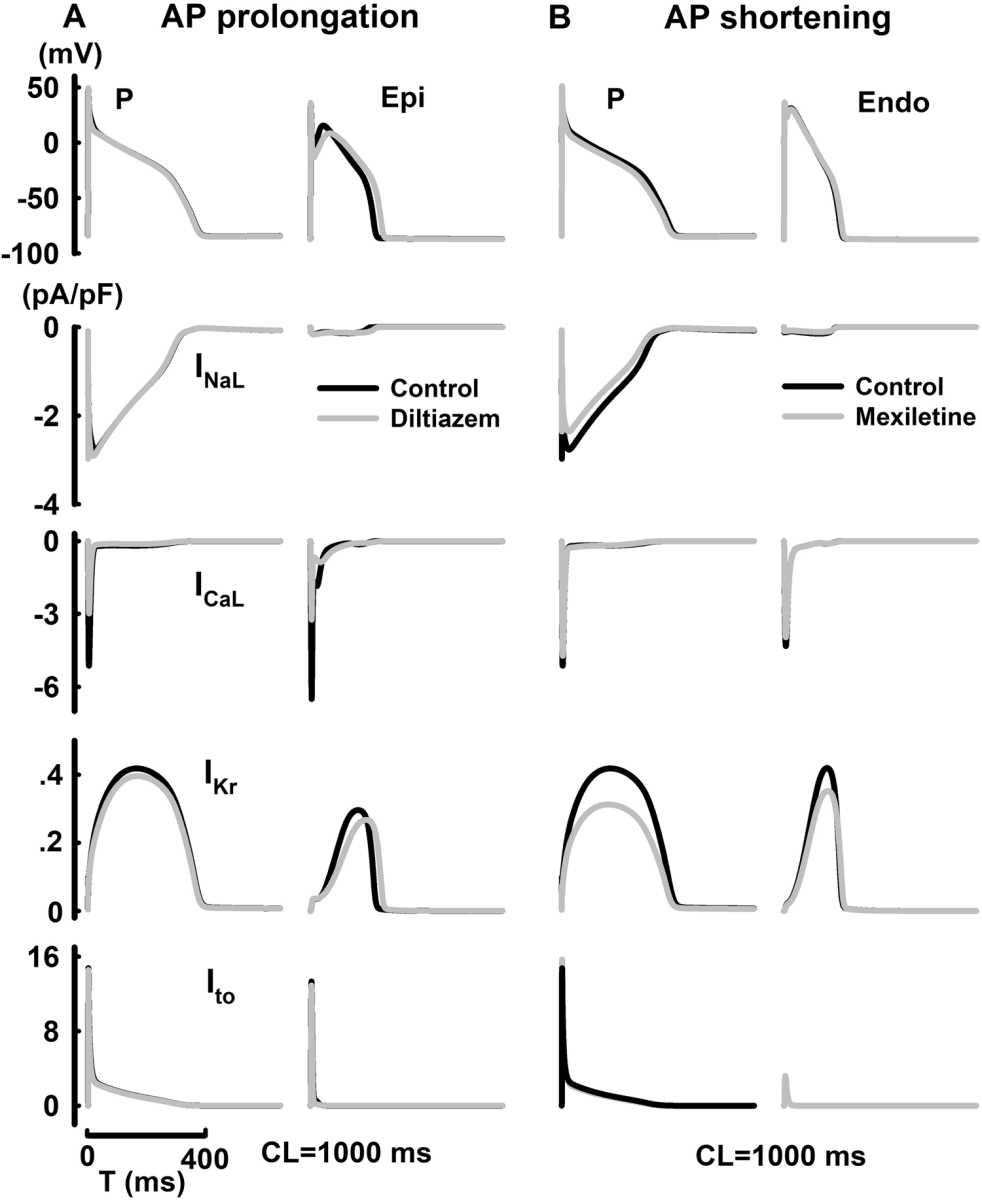
Steady-state cell-dependent AP prolongation, AP shortening and their underlying ionic currents (I_NaL_, I_CaL_, I_Kr_, and I_to_) with the application of diltiazem (**A**) or mexiletine (**B**).

**Figure 4:**
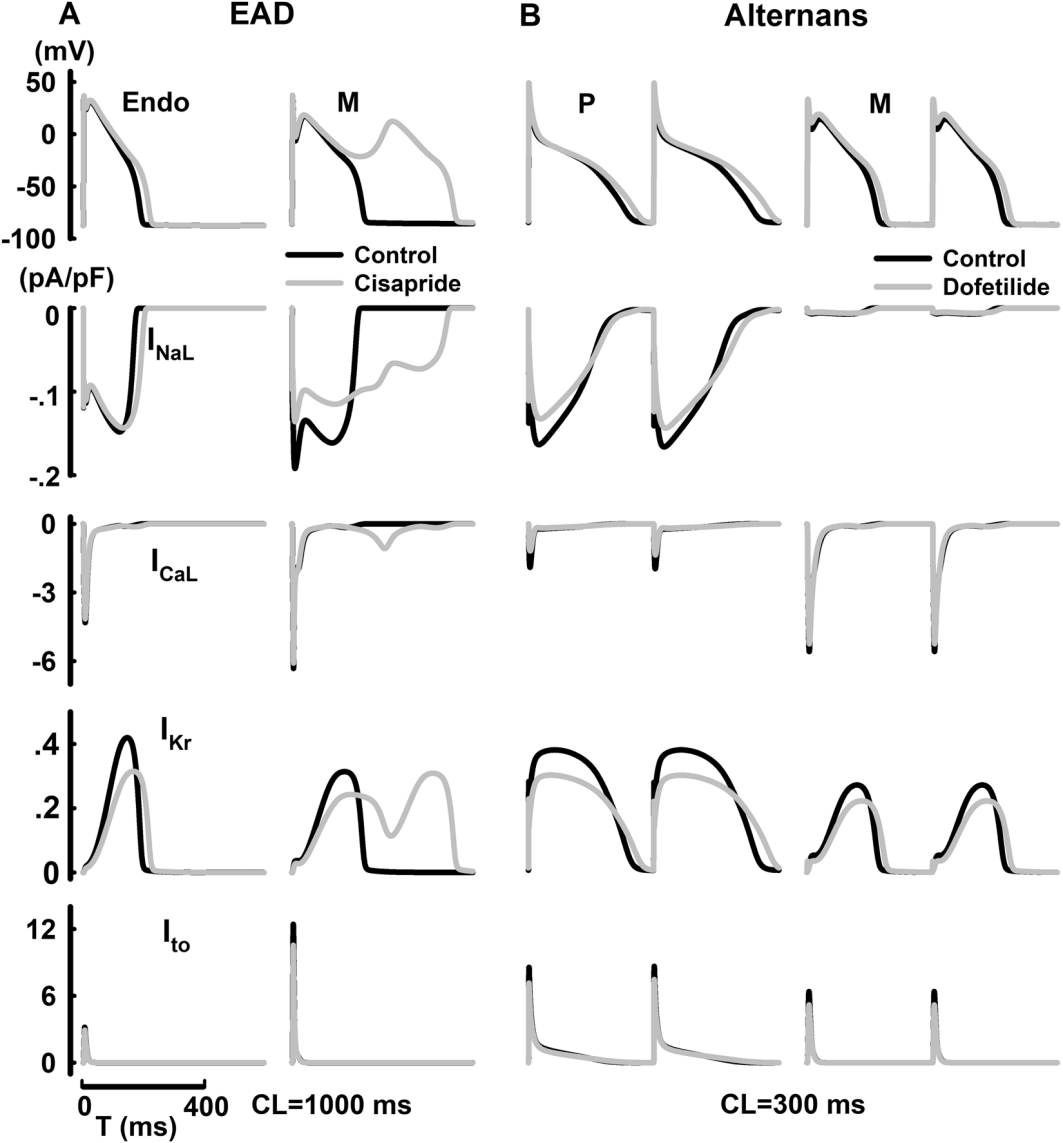
Steady-state cell-dependent EAD, AP alternans and their underlying ionic currents (I_NaL_, I_CaL_, I_Kr_, and I_to_) with the application of cisapride (**A**) or dofetilide (**B**).

The AP and ionic channel dynamics with the application of diltiazem and mexiletine were shown at CL = 1000 ms in Fig. 3. With the application of diltiazem, AP morphology and APD remain largely unaffected despite of a major reduction in I_CaL_ in P cells, while accentuated AP notch and APD prolongation were observed in Epi cells due to decreased I_CaL_ and I_Kr_. With the application of mexiletine, minor APD shortening or prolongation was evident in P or endo cells respectively. While I_Kr_ reduction promotes APD prolongation in both cell types, such effect was mitigated by the concurrent inhibition of Na^+^ channels in P cells. Transmural variations of drug-induced EAD and AP alternans were show in Fig. 4. With the application of cisapride (CL = 1000 ms), major reduction in I_Kr_ caused APD prolongation by 15.7% in Endo cells, yet induced EAD in M cells.With the application of dofetilide (CL = 300 ms), AP alternans was observed at CL = 300 ms most due to alternating large-small I_NaL_ currents in P cells, while no alternans was shown in M cells under same conditions.

### Rate-dependent variations of drug-induced arrhythmogenicity

**Figure 5:**
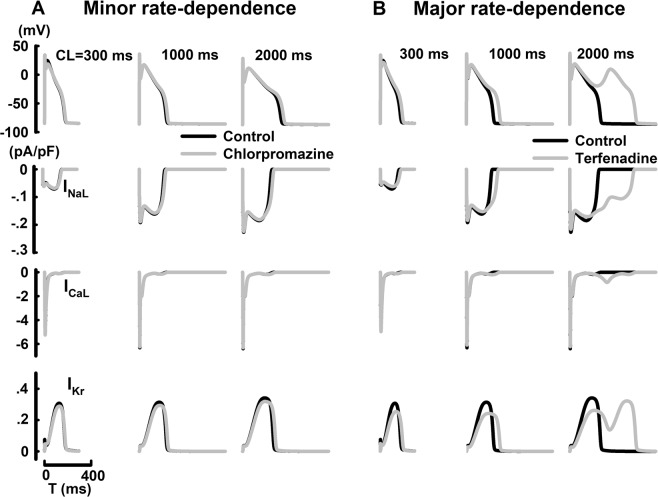
Steady-state rate-dependent variations and their underlying ionic currents (I_NaL_, I_CaL_, I_Kr_, and I_to_) with the application of chlorpromazine (**A**) or terfenadine **(B**) in M cells at different CLs.

AP and underlying ionic channel dynamics at different CLs (300 ms, 1000 ms and 2000 ms) with the application of chlorpromazine and terfenadine in M cells were shown in Fig. 5. The effects chlorpromazine exhibit minor rate-dependence with slightly pronounced APD prolongation at CL = 2000 ms, compared to CL = 300 ms. With the application of terfenadine, major APD prolongation at CL = 1000 ms (23.6%) was observed compared to CL = 300 ms. At CL = 2000 ms, terfenadine induced EAD due to the reactivation of I_CaL_. Compared to chlorpromazine, terfenadine displays significant rate-denpendence in M cells under same conditions.

### Drug-induced early afterdepolarization (EAD) and AP alternans

**Figure 6:**
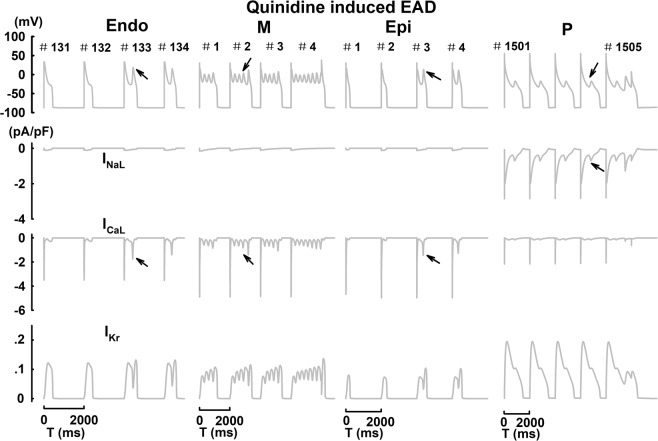
Qunidine induced EAD events and underlying ionic currents in Endo, M, Epi and P cells at CL = 2000 ms. Black arrows indicate EAD events.

**Figure 7:**
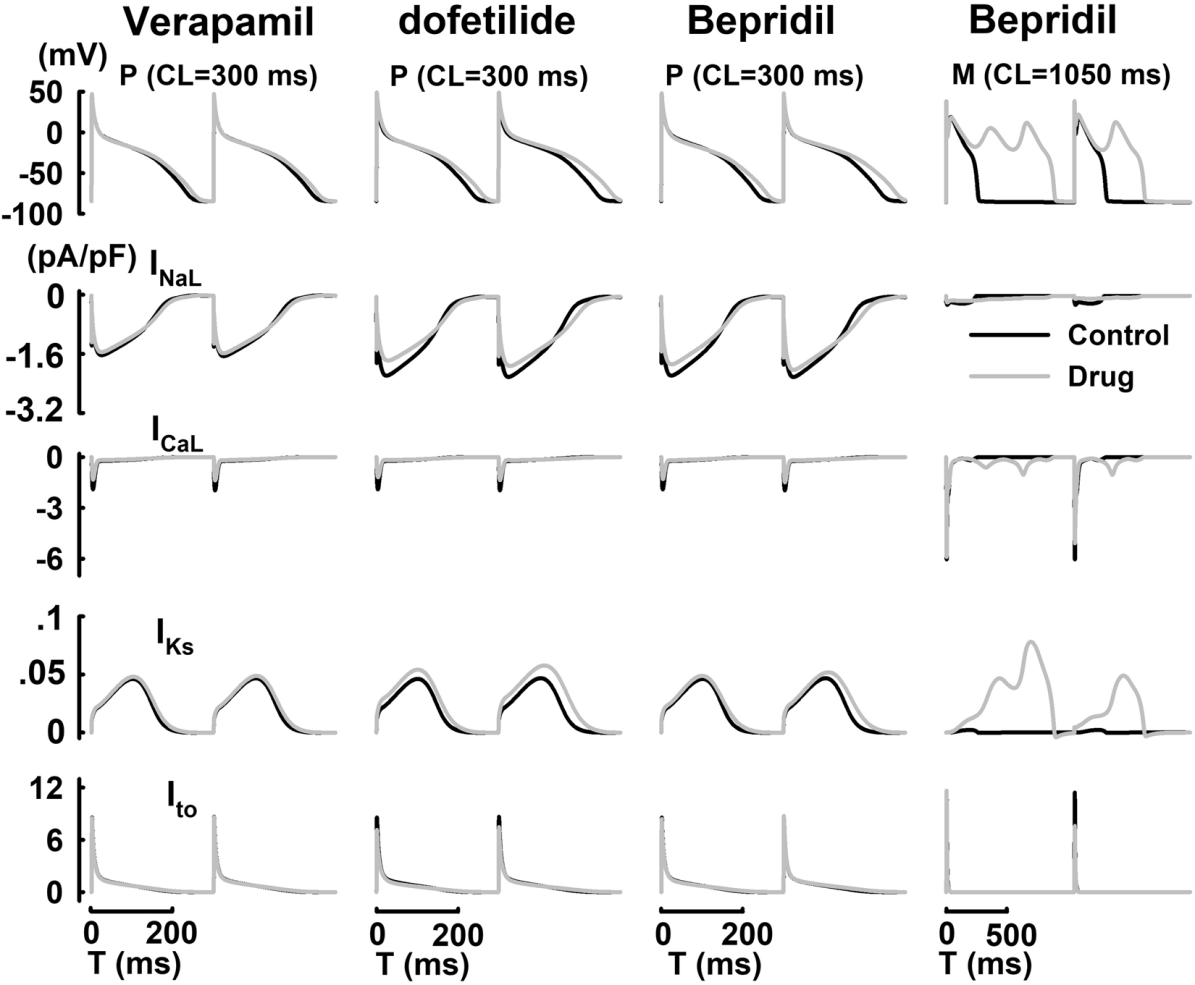
Steady-state AP alternans and underlying ionic currents with the application of verapamil, dofetilide or bepridil at various CLs.

With the application of quinidine at CL = 2000 ms, EAD events were observed in all cell types (Fig. 6). In ventricular cells (Endo, M and Epi), the reactivation of I_CaL_ is evident, and it coincides with the EAD upstroke. Particularly, oscillatory EAD events can been observed in M cells along with the repetitive reactivation of I_CaL_. However, in P cells, the reactivation of I_CaL_ is much smaller in amplitude, and contributes a secondary role in trigger EAD; the EAD upstroke is mostly driven by I_NaL_ due to intrinsic electrophysiological heterogeneities between Purkinje and ventricular cells^28^. Such observation is consistent with previous studies^28^. Drug-induced AP alternans and underlying ionic currents were shown in Fig. 7. With the application of dofetilide or bepridil, steady-state AP prolongation with enhanced beat-to-beat variations (AP alternans) was observed at CL = 300 ms in P cells; with the application of verapamil, AP prolongation with no beat-to-beat variations was shown^28^. In addition, the application of bepridil can also induce steady-state alternating EAD patterns (1 vs 2 EAD upstrokes from beat to beat) at slower pacing rates (CL = 1050 ms) in M cells. While I_NaL_ is mostly responsible to induce AP alternans in P cells at CL = 300 ms, I_CaL_ and I_to_ play such role in driving AP alternans associated with the EAD morphology in M cells at CL = 1050 ms.

## Discussion

In this study, we quantitatively evaluated the transmural characteristics and rate dependence of drug-induced arrhythmogenicity through simulations of multichannel pharmacology using a family of cardiac cell models. Our results reveal diverse cell- and rate-dependent variations of drug-induced arrhythmogenicity, e.g. AP alternans, APD shortening or prolongation, EAD events. For instance, the effects of mexiletine (low risk) exhibit little cell and rate-dependent variations, while significant cell and rate-dependence was observed with the application of bepridil (high risk). In addition, our simulation results suggest QT prolongation alone might be insufficient in predicting TdP risk, since major APD prolongation can be observed with the application of diltiazem or verapamil (low risk) with no EAD events. Veramamil inhibits both I_Kr_ and I_CaL_ currents, thus it can promote APD prolongation with a smaller I_CaL_ current incapable of triggering EAD events. Thus, in addition to QT prolongation alone, it appears that the occurrence and onset of EAD events, especially within physiological pacing window, could be a candidate biomarker for assessing TdP liability. A total of 12 drug were quantitatively evaluated in our study, and only quinidine (high risk) is capable of inducing EAD in all cell types. The predicted high TdP risk of quinidine is consistent with that in previous clinical studies^29^. Our simulation results with ranolazine (low risk) seems to be in conflict with its safety observed in clinical trials^30^, yet it should be noted that ranolazine induced EAD at CL >1800 ms (~33 beats per minute) in M cells. Considering electric coupling in the intact ventricular myocardium, ranolazine induced EAD events might occur at very slow and unphysiological pacing rates according to our simulations. However, our results might also indicate possible hidden risks associated with ranolazine. Furthermore, our predictions regarding chlopromazine and ondansetron (minor changes in APD rate adaptation with no EAD events) seem not well in line with their known intermediate risks. This is likely due to their relatively low EFTPC values and minor inhibition effects on ion channels. These results might also indicate possible drug targets other than the CiPA ion channels associated with chlopromazine and ondansetron.

Our simulation results suggest that M cells, in general, are much more vulnerable to both drug-induced AP prolongation and EAD occurrence, and the intrinsic arrhythmogenicity of M cells can be much higher than that of other cell types. Despite the controversies surrounding M cells, our results support a major role of the M cell in promoting pathologic heterogeneities of repolarization^31^. In addition to electric inter-cellular coupling, location and abundance of M cells in the intact ventricular myocardium could be important determinants to extrapolate our cell simulation results at tissue-level^31^. In addition, we found that drug-induced changes in APD adaptation can be important during the evaluation of drug-induced cardiotoxicity. The pronounced increase in APD at slower heart rates allows for the recovery of inactivated Ca^2+^ or Na^+^ channels, widening the window of EAD generation, a cellular event that can induce TdP^32–35^. Furthermore, we found that the mechanism of drug-induced EAD generation differs across cell types; EAD generation in P cells is mostly due to reactivation of I_NaL_, while in Endo, M and Epi cells, I_CaL_ plays the predominant role^28^. Intrinsic electrophysiological and ultrastructural heterogeneities between Purkinje and ventricular cells are most responsible for different AP morphologies and ionic mechanisms underlying AP rate adaptation, restitution and EAD events^28^. For instance, I_NaL_ in Purkinje cells is a much larger current with distinct decay and recovery kinetics, while I_CaL_ is much smaller compared to ventricular cells^36–38^. Purkinje cells are devoid of transverse tubular (T-tubular) network, and exhibits biphasic Ca^2+^ transient in response to membrane depolarization^39^. Compared to other ventricular cells, M cells are characterized with prolonged APD and less repolarization reserve mostly due to a larger I_NaL_ and a smaller I_Ks_, and are therefore predisposed to a higher risk of arrhythmogenesis. In addition, comparing to other cell types, P cells are generally more sensitive to drug-induced AP alternans at fast pacing rates, that may potentially lead to Purkinje-ventricular conduction abnormalities at tissue/organ level^40^.

We concluded that simulations of multichannel pharmacology in diverse cell types at all physiological pacing rates could be essential to evaluate drug-induced arrhythmogenic risks, and an updated and more comprehensive list of biomarkers associated with cell- and rate-dependent drug responses, instead of hERG inhibition or QT prolongation alone, might improve the prediction of drug-induced cardiotoxicity. Further model development is required to develop human-specific purkinje-ventricular tissue models with spatially distributed intra- and intercellular Ca^2+^ dynamics, to predict arrhythmogenic risks associated with abnormal Ca^2+^ handling, such as spontaneous Ca^2+^ release and delayed after-depolarization (DAD)^41^.

## Methods

A family of cardiac cell models (P, Endo, M and Epi) were derived from the Purkinje and ventricular cell models published by Li and Rudy^28^ and Decker *et al*.^42^ based on experimental measurements of intrinsic heterogeneities of electrophysiological properties (see Supplemental Table S2). These models were developed to include physiologically based representations of intracellular Ca^2+^ cycling and major membrane ionic currents specific to each cell types, using similar mathematical modelling and validation approaches^28,42^. Multi-channel inhibition effects of 12 CiPA training compounds with known TdP risk (including quinidine, bepridil, dofetilide, sotalol, chlorpromazine, cisapride, terfenadine, ondansetron, diltiazem, mexiletine, ranolazine and verapamil) were simulated at effective free therapeutic plasma concentration (EFTPC) (see Supplemental Table S3 for details)^43^; a more extensive list of compounds with known TdP risk classifications has been reported in previous studies^6,20,44^. Steady-state simulation results are for 60 minutes of pacing at a given CL. APD is determined as APD_90_ (90% repolarization). Standard programming language C was used. Forward Euler method with an adaptive time step was used for numeric integration. To compare cell-dependent responses, drug-induced changes in AP adaptation were quantified as the percentage of ΔAPD_90,drug_/APD_90,control_.

## Supplementary information

Supplementary information
