# Supplementary material for: Transmural and rate-dependent profiling of drug-induced arrhythmogenic risks through in silico simulations of multichannel pharmacology
Source: Sci Rep. 2019 Dec 6;9:18504. doi: 10.1038/s41598-019-55032-x (PMC6898675; doi:10.1038/s41598-019-55032-x)
Supplement: Supplementary file 1 — Supplementary information [file 41598_2019_55032_MOESM1_ESM.pdf]

Supplementary Materials

To simulate a drug blocking a channel, we used the following equation to scale G_x, based on the IC_{50} and concentration:

$$G_{x,drug} = G_x \left[1 + \left(\frac{C}{IC_{50,x}}\right)^h\right]^{-1}$$

where $G_{x,drug}$ is the maximal conductance of channel x in the presence of the drug; C is the concentration of the drug; $IC_{50,x}$ is the half-maximal inhibitory concentration for that drug; h is the Hill coefficient.

Table S1 A detailed summary of simulated drug-induced APD prolongation, EAD, AP alternans and reported TdP risks of 12 CiPA training compounds[1-3].

	**CiPA TdP Category**	**The onset of EAD**	**AP alternans (onset and magnitude)**	**ΔAPD% (CL=1000 ms)**
**Quinidine**	High	At all CLs in all cell types	Not applicable	P (91.39%); Epi (EAD); Endo (EAD); M (EAD)
**Bepridil**	High	CLs>550ms in M cell	At CLs=300~450ms in P cell (6.1817%) At CLs=950~1100ms in M cell (EAD and Alternans)	P (15.01%); Epi (27.02%); Endo (25.64%); M (EAD)
**Dofetilide**	High	At CLs>850ms in M cell	At CLs=300~450ms in P cell (6.574%)	P (15.15%); Epi (15.75%); Endo (17.73%); M (EAD)
**Sotalol**	High	At CLs>1550ms in M cell	At CLs=300~450ms in P cell (1.883%)	P (5.53%); Epi (7.75%); Endo (8.09%); M (12.26%)
**Chlorpromazine**	Intermediate	Not shown	At CLs=300~450ms in P cell (1.834%)	P (2.32%); Epi (3.36%); Endo (3.38%); M (4.69%)
**Cisapride**	Intermediate	At CLs>900ms in M cell	At CLs=300~450ms in P cell (2.744%)	P (12.64%); Epi (14.30%); Endo (15.71%); M (EAD)
**Terfenadine**	Intermediate	At CLs>1050ms In M cell	At CLs=300~450ms in P cell (1.949%)	P (9.73%); Epi (13.25%); Endo (13.69%); M (23.65%)
**Ondansetron**	Intermediate	Not shown	At CLs=300~450ms in P cell (1.852%)	P (3.55%); Epi (5.21%); Endo (5.23%); M (7.63%)
**Diltiazem**	Low/no risk	Not shown	At CLs=300~450ms in P cell (1.477%)	P (-0.53%); Epi (11.78%); Endo (0.94%); M (8.36%)
**Mexiletine**	Low/no risk	Not shown	At CLs=300~450ms in P cell (2.014%)	P (-2.29%); Epi (3.78%); Endo (2.63%); M (3.85%)
**Ranolazine**	Low/no risk	At CLs>1800ms in M cell	At CLs=300~450ms in P cell (1.815%)	P (0.92%); Epi (6.40%); Endo (6.88%); M (9.43%)
**Verapamil**	Low/no risk	Not shown	At CLs=300~450ms in P cell (0.793%)	P (7.18%); Epi (13.93%); Endo (10.43%); M (17.01%)

Table S2 Model details of Epi, Endo and M cells according to experimental measurements[4-6].

Cell Type	Scaling factor (Epi as control)			
**Epi**	1*G_{NaL}	1*G_{to1}	1*G_{Ks}	1*G_{NaCa}
**Endo**	1* G_{NaL}	0.18* G_{to1}	0.9* G_{Ks}	0.9* G_{NaCa}
**M**	1.4* G_{NaL}	0.91* G_{to1}	0.3* G_{Ks}	1.3* G_{NaCa}

Table S3 IC_{50} and Hill coefficients (h) of 12 CiPA training compounds[1,7].

**Drug**		**hERG (I_{Kr})**	**I_{NaL}**	**I_{CaL}**	**I_{Na}**	**I_{to}**	**I_{K1}**	**I_{Ks}**	**Free Cmax, nmol/L**
**Quinidine**	IC_{50} (nmol/L)	992	9417	51592	12329	3487.4	4E+07	4898.9	3237
	h	0.8	1.3	0.6	1.5	1.3	0.4	1.4	
**Bepridil**	IC_{50} (nmol/L)	50	1813.9	2808.1	2929.3	8594	-*	28628.3	33
	h	0.9	1.4	0.6	1.2	3.5	-	0.7	
**Dofetilide**	IC_{50} (nmol/L)	4.9	753160	260.3	380.5	18.8	394.3	-	2
	h	0.9	0.3	1.2	0.9	0.8	0.8	-	
**Sotalol**	IC_{50} (nmol/L)	110600	-	7E+06	1140000000	43143455	3E+06	4221856	14690
	h	0.8	-	0.9	0.5	0.7	1.2	1.2	
**Chlorpromazine**	IC_{50} (nmol/L)	929.2	4559.6	8191.9	4535.6	17616711	9269.9	-	38
	h	0.8	0.9	0.8	2	0.4	0.7	-	
**Cisapride**	IC_{50} (nmol/L)	10.1	-	9E+06	-	219112.4	29498	81192862	2.6
	h	0.7	-	0.4	-	0.2	0.5	0.3	
**Terfenadine**	IC_{50} (nmol/L)	23	20056	700.4	4803.2	239960.8	-	399754	4
	h	0.6	0.6	0.7	1	0.3	-	0.5	
**Ondansetron**	IC_{50} (nmol/L)	1320	19181	22551	57666.4	1023378	-	569807	139
	h	0.9	1	0.8	1	1	-	0.7	
**Diltiazem**	IC_{50} (nmol/L)	13150	21869	112.1	110859	2820000000	-	-	122
	h	0.9	0.7	0.7	0.7	0.2	-	-	
**Mexiletine**	IC_{50} (nmol/L)	28880	8956.8	38244	-	-	-	-	4129
	h	0.9	1.4	1	-	-	-	-	
**Ranolazine**	IC_{50} (nmol/L)	8270	7884.5	-	68774	-	-	36155020	1948.2
	h	0.9	0.9	-	1.4	-	-	0.5	
**Verapamil**	IC_{50} (nmol/L)	288	7028	201.8	-	13429.2	3E+08	-	81
	h	1	1	1.1	-	0.8	0.3	-	

*- indicates no detectable blocking for that channel.

Reference

1 Li, Z. *et al.* Improving the In Silico Assessment of Proarrhythmia Risk by Combining hERG (Human Ether-a-go-go-Related Gene) Channel-Drug Binding Kinetics and Multichannel Pharmacology. *Circ Arrhythm Electrophysiol* 10, e004628, doi:10.1161/CIRCEP.116.004628 (2017).

2 Colatsky, T. *et al.* The Comprehensive in Vitro Proarrhythmia Assay (CiPA) initiative - Update on progress. *J Pharmacol Toxicol Methods* 81, 15-20, doi:10.1016/j.vascn.2016.06.002 (2016).

3 Fermini, B. *et al.* A New Perspective in the Field of Cardiac Safety Testing through the Comprehensive In Vitro Proarrhythmia Assay Paradigm. *J Biomol Screen* 21, 1-11, doi:10.1177/1087057115594589 (2016).

4 Zygmunt, A. C., Eddlestone, G. T., Thomas, G. P. & et, a. l. Larger late sodium conductance in M cells contributes to electrical heterogeneity in canine ventricle. *Am J Physiol Heart Circ Physiol* 281:H689-97. (2001).

5 Murphy, L., Renodin, D., Antzelevitch, C., Di Diego, J. M. & Cordeiro, J. M. Extracellular proton depression of peak and late Na(+) current in the canine left ventricle. *Am J Physiol Heart Circ Physiol* 301, H936-944, doi:10.1152/ajpheart.00204.2011 (2011).

6 Benson, A. P., Aslanidi, O. V., Zhang, H. & Holden, A. V. The canine virtual ventricular wall: a platform for dissecting pharmacological effects on propagation and arrhythmogenesis. *Prog Biophys Mol Biol* 96, 187-208, doi:10.1016/j.pbiomolbio.2007.08.002 (2008).

7 Crumb, W. J., Jr., Vicente, J., Johannesen, L. & Strauss, D. G. An evaluation of 30 clinical drugs against the comprehensive in vitro proarrhythmia assay (CiPA) proposed ion channel panel. *J Pharmacol Toxicol Methods* 81, 251-262, doi:10.1016/j.vascn.2016.03.009 (2016).
